# A Novel Assay for the Identification of NOTCH1 PEST Domain Mutations in Chronic Lymphocytic Leukemia

**DOI:** 10.1155/2016/4247908

**Published:** 2016-12-15

**Authors:** Paulo Vidal Campregher, Roberta Cardoso Petroni, Nair Hideko Muto, Roberta Sitnik, Flavia Pereira de Carvalho, Nydia Strachman Bacal, Elvira Deolinda Rodrigues Pereira Velloso, Gislaine Borba Oliveira, João Renato Rebello Pinho, Davi Coe Torres, Marcela Braga Mansur, Rocio Hassan, Irene Gyongyvér Heidemarie Lorand-Metze, Carlos Sérgio Chiattone, Nelson Hamerschlak, Cristovão Luis Pitangueira Mangueira

**Affiliations:** ^1^Departments of Hematology and Clinical Pathology, and Research Institute, Hospital Israelita Albert Einstein, São Paulo, SP, Brazil; ^2^Department of Hematology, Universidade Estadual de Campinas (Hemocentro-Unicamp), Campinas, SP, Brazil; ^3^Department of Clinical Pathology, Hospital Israelita Albert Einstein, São Paulo, SP, Brazil; ^4^Hematology Service, Hospital das Clínicas, Faculdade de Medicina da Universidade de São Paulo São Paulo, SP, Brazil; ^5^Cytogenetics Laboratories, Hospital Israelita Albert Einstein, São Paulo, SP, Brazil; ^6^Hemocentro, Faculdade de Medicina da Universidade Estadual de Campinas (Hemocentro-Unicamp), Campinas, SP, Brazil; ^7^Bone Marrow Transplantation Center, Instituto Nacional de Câncer (INCA), Rio de Janeiro, RJ, Brazil; ^8^Paediatric Haematology-Oncology Program, Instituto Nacional de Câncer (INCA), Rio de Janeiro, RJ, Brazil; ^9^Department of Hematology, Faculdade de Medicina da Santa Casa de Misericórdia de São Paulo, São Paulo, SP, Brazil; ^10^Department of Hematology, Hospital Israelita Albert Einstein, São Paulo, SP, Brazil

## Abstract

*Aims.* To develop a fast and robust DNA-based assay to detect insertions and deletions mutations in exon 34 that encodes the PEST domain of* NOTCH1* in order to evaluate patients with chronic lymphocytic leukemia (CLL).* Methods.* We designed a multiplexed allele-specific polymerase chain reaction (PCR) combined with a fragment analysis assay to detect specifically the mutation c.7544_7545delCT and possibly other insertions and deletions in exon 34 of* NOTCH1*.* Results.* We evaluated our assay in peripheral blood samples from two cohorts of patients with CLL. The frequency of* NOTCH1* mutations was 8.4% in the first cohort of 71 unselected CLL patients. We then evaluated a second cohort of 26 CLL patients with known cytogenetic abnormalities that were enriched for patients with trisomy 12.* NOTCH1* mutations were detected in 43.7% of the patients with trisomy 12.* Conclusions.* We have developed a fast and robust assay combining allele-specific PCR and fragment analysis able to detect* NOTCH1* PEST domain insertions and deletions.

## 1. Introduction

Chronic lymphocytic leukemia (CLL) is a neoplastic disorder of B lymphocytes characterized by progressive increase in CD5+ B cells in the peripheral blood (PB), bone marrow, and lymph nodes. The clinical course of CLL is highly heterogeneous, with some patients experiencing indolent disease, with no treatment requirement for several years, and others showing aggressive course and decreased overall survival (OS) [1].

Although several prognostic factors have been identified in CLL, such as* TP53* mutation, cytogenetic abnormalities, IGVH mutational status, the only biomarkers utilized to inform clinical decisions nowadays are 17p or* TP53* mutations [1]. Since these alterations are present in a minority of patients, most CLL cases are treated based on clinical criteria.

Recently, novel prognostic relevant recurrent mutations in genes such as NOTCH1, SF3B1, and BIRC3 have been identified in CLL [2, 3]. The most common of these alterations are* NOTCH1* and* SF3B1* mutations, which are present in around 10% of CLL patients at diagnosis [2].

*NOTCH1* mutations in CLL occur almost exclusively in the genic region encoding the PEST domain and are characterized by frameshift insertions and deletions, with the mutation c.7544_7545delCT representing more than 75% of the mutations [4, 5]. In addition,* NOTCH1* mutations have been associated with decreased OS and treatment-free survival (TFS) in several independent studies [3–6]. The goal of this work was to develop a simple, robust, and cost-effective assay to identify* NOTCH1* PEST domain mutations and also to evaluate the frequency of* NOTCH1* mutations in a cohort of CLL patients.

## 2. Methods

This study was approved by the Institutional Review Board of Hospital Israelita Albert Einstein (protocol 1699-13). Consent form was waived, since the samples were deidentified and the researchers did not have access to clinical information. Peripheral blood mononuclear cells were isolated (PBMC) by Ficoll-Paque gradient centrifugation and genomic DNA was extracted with QIAamp DNA Mini Kit (Qiagen). Fragment analysis and sequencing with BigDye Terminator v3.1 Cycle Sequencing Kit were performed in the equipment 3500XL Genetic Analyzer (Applied Biosystems).

Polymerase chain reaction (PCR) amplification and sequence analysis of* IGHV-D-J* rearrangements were performed on genomic DNA from PB samples, using VH framework region 1 (FR1) consensus family specific primers (VH1–VH6) and a JH primer, according to the BIOMED-2 protocol [7]. When amplification was unsuccessful, an alternative set of primers that anneal to the leader region (LH1–LH6) and a JH consensus primer were used. Clonal PCR products were purified using Wizard SV Gel and PCR Clean-Up System (Promega), and both strands were directly sequenced with BigDye Terminator v3.1 Cycle Sequencing Kit (Applied Biosystems, Carlsbad, CA) on a 3130XL Genetic Analyzer (Applied Biosystems). The sequences were aligned to the closest matching germ line gene by using IMGT's V-QUEST analysis tool (http://www.imgt.org/IMGT_vquest/share/textes/) and NCBI's IgBLAST tool (https://www.ncbi.nlm.nih.gov/igblast/). Sequences with a germ line identity greater than or equal to 98% were considered unmutated and those with an identity less than 98% were considered mutated [8].

Exact Fisher's test was used to compare patients with mutated and wild-type* NOTCH1* regarding* IGVH* mutational status.

## 3. Results

**Figure 1:**
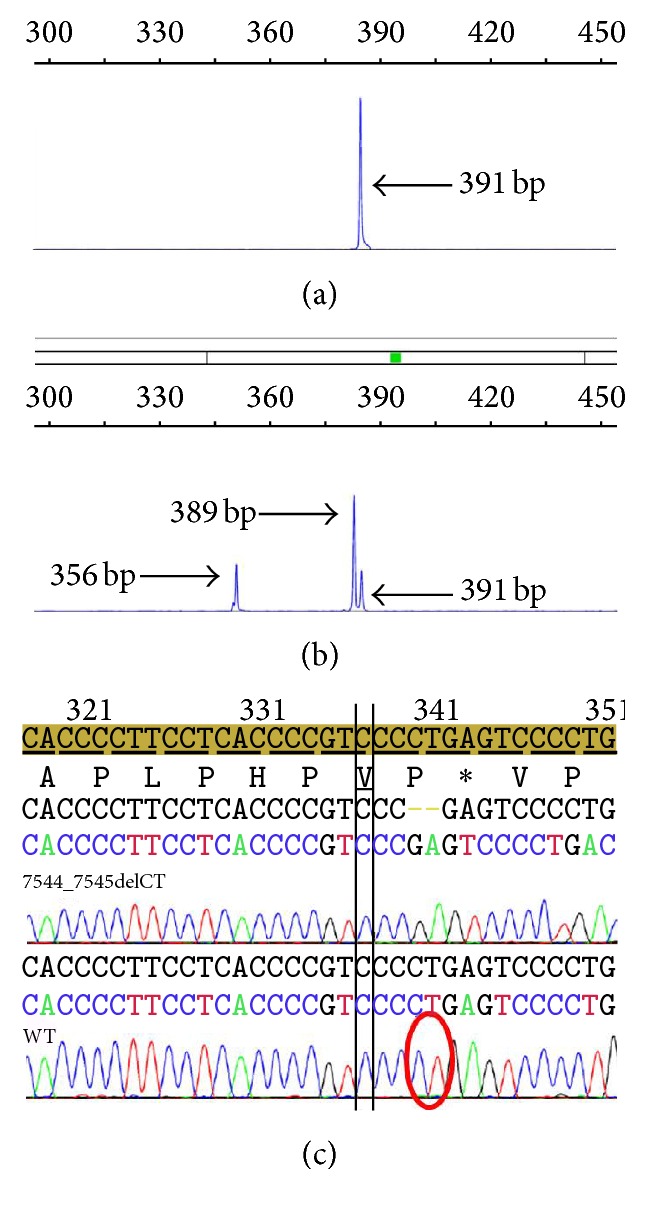
Fragment analysis and sequencing results. (a) Fragment analysis of a sample with wild-type NOTCH1 revealing a single 391 bp peak. (b) Fragment analysis of a chronic lymphocytic leukemia (CLL) sample harboring the NOTCH1 PEST domain mutation c.7544_7545delCT showing three distinct peaks: a 391 bp peak, corresponding to wild-type NOTCH1, a 389 bp peak, amplified with primers N1F and NWR (non-mutation-specific), revealing a 2 bp deletion, and a 356 bp peak, amplified with the allele-specific primer NMR. (c) Sanger sequencing of a sample carrying the NOTCH1 mutation c.7544_7545delCT (upper panel) and a wild-type sample (lower panel).

The assay we developed had two main goals: to detect insertions and deletions in the region of* NOTCH1* exon 34 encoding the PEST domain and also to specifically detect the mutation c.7544_7545delCT, which accounts for around 80% of all* NOTCH1* mutations in CLL [4]. To accomplish this, we designed a fragment analysis PCR with three primers: a forward primer (N1F) labeled with FAM (CATCCAGCAGCAGCAAAGC), a nonspecific reverse primer (NWR) (GTCGGAGACGTTGGAATGC) that amplifies the wild-type sequence, and another allele-specific reverse primer that amplifies only the mutation c.7544_7545delCT (NMR) (CCACTGGTCAGGGGACTCG). We then performed a single PCR reaction with the three primers. Samples with wild-type* NOTCH1* would produce a single product of 391 bp; samples harboring heterozygous c.7544_7545delCT would generate three products: 391 bp (wild-type allele), 389 bp (amplification of the mutated allele with primers N1F and NWR), and 356 bp (amplification of the mutated allele with N1F and NMR) (Figures 1(a) and 1(b)). Finally, samples with another heterozygous insertion or deletion in the PEST domain would yield two products, 391 bp from the wild-type allele and another variable product, depending on the size of the insertion or deletion.

To validate our assay, we evaluated two groups of CLL patients. The initial group consisted of consecutive deidentified samples from 71 patients with CLL in distinct disease stages that had PB flow cytometry evaluation at our laboratory, for which we had no clinical or cytogenetic information. We identified* NOTCH1* mutations in 6 patients (8.4%). To confirm that our test was indeed detecting the expected mutations, we performed Sanger sequencing of all 6 positive cases and 3 negative cases, confirming the specificity of our assay (Figure 1(c)).

We then analyzed a second group of 26 consecutive CLL patients that had been successfully evaluated with fluorescent in situ hybridization (FISH) at our laboratory. All these patients had recurrent cytogenetic abnormalities (trisomy 12, 13q, 17p, and 11q present in 16, 6, 3, and 1 patient, resp.). This group was enriched for patients with trisomy 12, since this population is known to harbor* NOTCH1* mutations frequently [9]. We found* NOTCH1* mutations in 10 patients of the second cohort (38.4%). Among patients with trisomy 12 (*n* = 16), the prevalence of* NOTCH1* mutations was 43.7% (*n* = 7), in agreement with previous reports [9]. All mutations identified in these patients (*n* = 16) were c.7544_7545delCT. We did not find other* NOTCH1* mutations in CLL patients. While 15 patients had heterozygous mutation, 1 patient presented with a homozygous mutation.

Since the presence of* NOTCH1* mutations has been associated with unmutated* IGVH* [5], we performed* IGVH* mutational status analysis in 63 out of the 97 analyzed patients, based on DNA availability. Among these samples, 9 had* NOTCH1* mutation and 54 had wild-type* NOTCH1*.* IGVH* mutational status was inconclusive in 10 patients due to low numbers of CLL cells in PB. Among the 53 evaluable patients, unmutated* IGVH* was detected in 100% of* NOTCH1* mutated cases (*n* = 7) and in 37% (*n* = 17) of patients with wild-type* NOTCH1* (*p* = 0.002), confirming previous findings [10].

We next determined the sensitivity of our test with serial dilutions of the cell line MOLT4 which carries a heterozygous c.7544_7545delCT* NOTCH1* mutation, using normal peripheral blood mononuclear cells. We diluted samples in the following way: 1 : 2, 1 : 5, 1 : 10, 1 : 20, and 1 : 40. The sensitivity of our test was 5%, meaning we can detect* NOTCH1* mutations when 5% of cells harboring* NOTCH1* heterozygous mutations are mixed with 95% of cells with wild-type* NOTCH1*.

**Figure 2:**
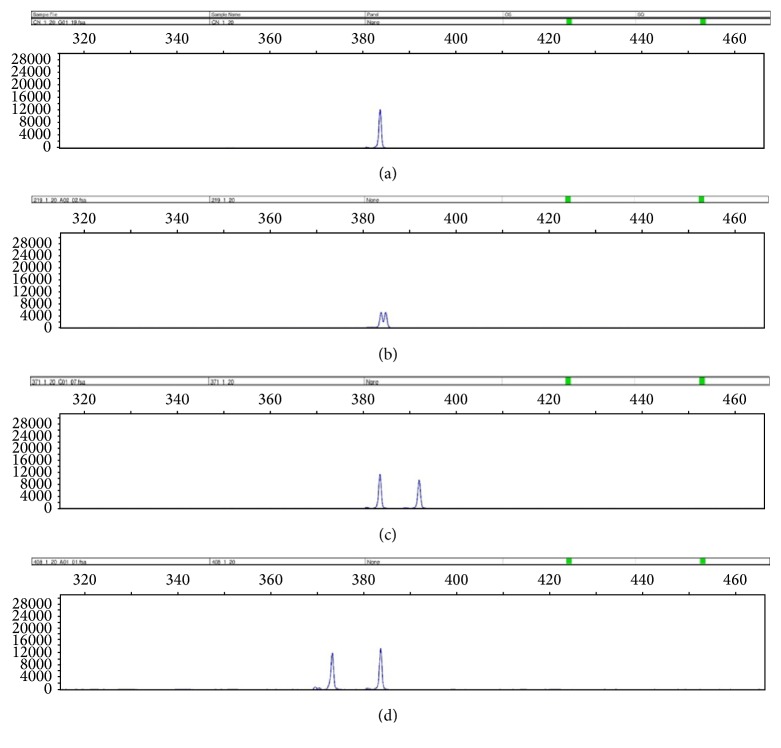
Fragment analysis of 4 samples not harboring the mutation c.7544_7545delCT. (a) Sample with wild-type NOTCH1 revealing a single 391 bp peak. (b) T-ALL140 sample harboring the mutation c.7526_7527insT, showing a 391 bp peak and a 392 bp peak. (c) T-ALL151 sample with the complex mutation c.7458_7464delGCAGCAC, c.7457_7458insAGGCGTCTAGCCGCAT, revealing a 391 bp peak and a 400 bp peak. (d) T-ALL176 sample with the mutation c.7529_7535delTCACCCC, depicting a 391 bp peak and a 384 bp peak.

In order to calculate the sensitivity of our assay to other exon 34* NOTCH1* insertions and deletions, given that the only mutation detected in CLL patients in our study was c.7544_7545delCT, we evaluated three samples from patients with T-acute lymphoblastic leukemia (T-ALL), known to harbor insertions and deletions in* NOTCH1* exon 34. Our test correctly identified all three mutations in these samples (see Figure 2).

## 4. Discussion

Mutated* NOTCH1* is a biomarker associated with Richter transformation, chemoresistance, and reduced overall and progression-free survival in CLL [10, 11]. These mutations are characterized by frameshift insertions and deletions in exon 34, with c.7544_7545delCT occurring in around 80% of mutated cases [4, 10]. The functional consequence of such* NOTCH1* mutations is the disruption of the PEST domain and stabilization of the* NOTCH1* protein leading to constitutive activation of* NOTCH1* pathway [12]. For these reasons, the evaluation of* NOTCH1* mutational status may have implications for the clinical management of CLL patients. We have thus developed a fast, robust, and sensitive assay for detection of* NOTCH1* PEST domain insertions and deletions.

Our data is consistent with previous findings of around 10% prevalence of* NOTCH1* mutations in unselected CLL patients and around 40% prevalence in CLL with trisomy 12 [6, 9]. Of note, mutations in the gene FBXW7, which belongs to NOTCH1 pathway, also seem to be more prevalent in patients with trisomy 12 and are associated with a worse outcome [13]. In addition, the previously described high correlation between unmutated* IGVH* and* NOTCH1* mutations was confirmed in our cohort [4, 10].

The identification of* NOTCH1* mutations in CLL can be useful as a prognostic marker [4] and also as a potential therapeutic target in the future [14]. The analysis of* NOTCH1* mutations in patients from the German CLL8 trial suggests that CLL patients harboring* NOTCH1* mutations may not benefit from the addition of rituximab to fludarabine and cyclophosphamide, indicating that* NOTCH1* may also become a predictive marker of response to rituximab in CLL [15].

While our test was developed to detect any insertions and deletions in exon 34, in our CLL cohort, we only identified the most common deletion, c.7544_7545delCT, in agreement with previous findings [3, 7]. Nevertheless, the identification of other insertions and deletions in T-ALL samples validates our assay to other mutations that are rarer and may be found in CLL (see Figure 2). Our assay does not detect single nucleotide variations that may be found on NOTCH1 exon 34; nevertheless, the pathogenic significance of such mutations remains to be defined [16].

In conclusion, we have developed a simple and robust assay combining allele-specific PCR and fragment analysis for the identification of* NOTCH1* PEST domain mutations. We have also shown that, among Brazilian patients analyzed, the prevalence of* NOTCH1* mutations and the correlation with unmutated* IGVH* in CLL are in agreement with previous reports [3–6].

## References

[1] Stilgenbauer S., Zenz T. (2010). Understanding and managing ultra high-risk chronic lymphocytic leukemia. *Hematology / the Education Program of the American Society of Hematology*.

[2] Campregher P. V., Hamerschlak N. (2014). Novel prognostic gene mutations identified in chronic lymphocytic leukemia and their impact on clinical practice. *Clinical Lymphoma, Myeloma and Leukemia*.

[3] Fabbri G., Rasi S., Rossi D. (2011). Analysis of the chronic lymphocytic leukemia coding genome: role of NOTCH1 mutational activation. *The Journal of Experimental Medicine*.

[4] Rossi D., Rasi S., Fabbri G. (2012). Mutations of NOTCH1 are an independent predictor of survival in chronic lymphocytic leukemia. *Blood*.

[5] Puente X. S., Pinyol M., Quesada V. (2011). Whole-genome sequencing identifies recurrent mutations in chronic lymphocytic leukaemia. *Nature*.

[6] Del Giudice I., Rossi D., Chiaretti S. (2012). NOTCH1 mutations in +12 chronic lymphocytic leukemia (CLL) confer an unfavorable prognosis, induce a distinctive transcriptional profiling and refine the intermediate prognosis of +12 CLL. *Haematologica*.

[7] van Dongen J. J. M., Langerak A. W., Brüggemann M. (2003). Design and standardization of PCR primers and protocols for detection of clonal immunoglobulin and T-cell receptor gene recombinations in suspect lymphoproliferations: report of the BIOMED-2 concerted action BMH4-CT98-3936. *Leukemia*.

[8] Hamblin T. J., Davis Z., Gardiner A., Oscier D. G., Stevenson F. K. (1999). Unmutated Ig V(H) genes are associated with a more aggressive form of chronic lymphocytic leukemia. *Blood*.

[9] Balatti V., Bottoni A., Palamarchuk A. (2012). NOTCH1 mutations in CLL associated with trisomy 12. *Blood*.

[10] Fabbri G., Rasi S., Rossi D. (2011). Analysis of the chronic lymphocytic leukemia coding genome: role of NOTCH1 mutational activation. *Journal of Experimental Medicine*.

[11] Nabhan C., Raca G., Wang Y. L. (2015). Predicting prognosis in chronic lymphocytic leukemia in the contemporary era. *JAMA Oncology*.

[12] Arruga F., Gizdic B., Serra S. (2014). Functional impact of NOTCH1 mutations in chronic lymphocytic leukemia. *Leukemia*.

[13] Falisi E., Novella E., Visco C. (2014). B-cell receptor configuration and mutational analysis of patients with chronic lymphocytic leukaemia and trisomy 12 reveal recurrent molecular abnormalities. *Hematological Oncology*.

[14] Groth C., Fortini M. E. (2012). Therapeutic approaches to modulating Notch signaling: current challenges and future prospects. *Seminars in Cell and Developmental Biology*.

[15] Stilgenbauer S., Schnaiter A., Paschka P. (2014). Gene mutations and treatment outcome in chronic lymphocytic leukemia: results from the CLL8 trial. *Blood*.

[16] Weissmann S., Roller A., Jeromin S. (2013). Prognostic impact and landscape of NOTCH1 mutations in chronic lymphocytic leukemia (CLL): a study on 852 patients. *Leukemia*.

